# Development of modern immunization agent against bovine papillomavirus type 1 infection based on BPV1 L1 recombinant protein

**DOI:** 10.3389/fvets.2023.1116661

**Published:** 2023-03-28

**Authors:** Alena Vrablikova, Veronika Brezani, Ivan Psikal, Martin Fraiberk, Marek Sebela, Martina Fojtikova, Pavel Kulich, Renata Hezova, Josef Masek

**Affiliations:** ^1^Department of Pharmacology and Toxicology, Veterinary Research Institute, Brno, Czechia; ^2^Dyntec spol s. r.o., Terezin, Czechia; ^3^Faculty of Science, Charles University, Prague, Czechia; ^4^Department of Biochemistry, Faculty of Science, Palacký University, Olomouc, Czechia

**Keywords:** papillomavirus, recombinant, vaccination, chimeric protein, virus-like particles (VLPs)

## Abstract

Bovine papillomavirus type 1 L1 protein was produced in a baculovirus expression system and purified as virus-like particles (VLPs) by affinity chromatography using lectins. The morphological integrity of VLPs was confirmed by electron microscopy. Differences between the two detected variants were deciphered by mass spectrometry of peptides (MALDI-TOF). Mice were immunized with purified VLPs in doses of 10, 25, or 50 μg in combination with 1% saponin and 15% alhydrogel per dose as adjuvants. Analysis of the humoral immune response revealed increased levels of specific antibodies detected 3 weeks after the first immunization in all groups of animals. This was further significantly increased by the booster applied 3 weeks after the first dose, with the best immune response in a group of mice immunized by the largest dose of antigen. BPV1 L1 VLPs purified by affinity chromatography using lectins could be used for prophylactic immunization in veterinary medicine.

## Introduction

Papillomaviruses (PVs) represent a large family of small, non-enveloped, and double-stranded DNA viruses capable of infecting the epithelial cells of humans and animals. These viruses are host specific with stringent tropism for cutaneous cells and mucosal epithelial cells. In bovine animals, infection with *Bos taurus papillomavirus* (BPV) leads to the formation of benign cutaneous fibropapillomas, which regress in response to cell-mediated immunity (1, 2). In some cases, especially in immunosuppressed cows, it progresses to squamous cell carcinoma (3). In horses, infection with BPV can lead to locally aggressive single or multiple sarcoid lesions with rare regression (4). There is no association between the BPV type and the anatomic localization or macroscopic characteristics of the lesions (5).

The immune response against BPV infection is poor, probably due to the restriction of the virus life cycle to the epithelium where contact with the immune system is limited (3). The major L1 and minor L2 capsid proteins play a crucial role in the recognition of viruses by the immune system (6). Effective prophylactic vaccination against bovine papillomatosis and sarcoids in equines is still not available. Virus-like particles represent a promising vaccine platform for a diverse array of viruses. These particles mimic morphological and immunological features of native virions lacking the ability to replicate and causing disease due to the absence of a viral genome (7). Here, we describe the production of BPV1 L1 as VLPs in a baculovirus expression vector system (BEVS) and the purification of these particles by Lentil Lectin-Sepharose 4B as a promising method for the preparation of a prophylactic vaccine applicable in veterinary medicine.

## Materials and methods

### Preparation and expression of recombinant baculovirus with BPV1 L1 protein

Bovine papillomavirus was obtained from organ suspensions of bovine cutaneous papilloma by ultracentrifugation in a cesium chloride density gradient (8). Total DNA was isolated from tissue homogenate and used as the source of the L1 capsid protein gene, which was amplified by PCR, and cleavage sites for restriction enzymes EcoRI (CTTGAT**GAATTC**ATGGCGTTGTGGCAACAAGGCCAG) and PstI (CTTGAT**CTGCAG**TTATTTTTTTTTTTTTTTTGCAGGC TTACTGG) were introduced to the ends of the L1 gene using primers. The gene coding L1 protein was inserted entirely using overlays detected by BLAST. MF384289.1 is the accession number of the sequence used for designing the primers.

The expression laboratory strain of baculovirus AcMNPV was generated using the Bac-to-Bac system (Thermo Fisher, USA). The amplified gene was inserted into linearized and alkaline phosphatase (Fermentas) dephosphorylated pFastBac1 vector, which was transformed into *Escherichia coli* DH10Bac bacteria grown in LB medium with 50 μg/ml kanamycin, 7 μg/ml gentamicin, and 10 μg/ml tetracycline. Recombinant baculovirus DNA was isolated using the PureLink™ HiPure Plasmid DNA Miniprep kit (Thermo Fisher Scientific) and transfected into Sf9 cells by lipofection (Cellfectin II reagent, Thermo Fisher Scientific). Recombinant baculoviruses released into the growth media (V_0_) were harvested 72 h after transfection.

Insect Sf9 cells (Gibco, USA) were grown in suspension culture at 27.5°C in SFM900II medium (Gibco) supplemented with gentamicin (50 μg/ml) in conical TubeSpin Bioreactors (TPP, Switzerland) using a temperature-controlled shaker. A total of 2.5–3 × 10^7^ Sf9 cells were infected with V_0_ at 0.1 MOI by shaking at 200 rpm to generate a high-titer viral stock. The supernatant (V_1_) was collected after 4 days of cultivation and used for infection of other 2.5–3 × 10^7^ cells in 10 ml SFM900II medium at 5 MOI. The cell culture was cultivated for 3 days at 27°C by shaking at 200 rpm, and the supernatant (V_2_) was used to infect 3 × 10^8^ cells in 150 ml SFM900II medium in 600 ml TPP^®^ TubeSpin bioreactor bottles for over-expression of BPV1 L1 VLPs for 156 h by shaking at 200 rpm. Sf9 cells were harvested, and the pellet was frozen at −80°C overnight.

### Purification of BPV1 L1 protein as VLPs

The cell pellet was resuspended in native lysis buffer (20° mmol°l^−1^ Tris-HCl, 1°mol l^−1^ NaCl, pH 7.6), supplemented with complete™, an EDTA-free Protease Inhibitor Cocktail (Merck, USA), lysed by sonication 3 × 30 s with 50% amplitude by sound MS73 in Sonoplus Ultrasonic Homogenizer (Bandelin, Germany), and cooled using ice. Cell lysates were centrifuged at 10,500 *g* for 10 min, the supernatant was diluted using 20 mmol l^−1^ Tris-HCl, pH 7.6 in a ratio of 1:2, and the mixture was treated using 180 U DENARASE (c-LEcta GmbH, Germany) and 2 μl 1 M MgCl_2_ per ml of the mixture. The sample was incubated for 1 h at 37°C by shaking and centrifuged at 10,500 g for 10 min. VLPs from the supernatant were purified using Lentil Lectin-Sepharose^®^ 4B (Cytiva, Czech Republic), according to the manufacturer's instructions. The cell lysate and purified protein were separated on 10% SDS-PAGE stained with Coomassie Blue R-250 or blotted onto a polyvinylidene difluoride (PVDF) membrane (Millipore, USA). The PVDF membrane blocked overnight with 3% non-fat milk was developed with anti-BPV1 L1 primary antibody (monoclonal antibody ab 2417, Abcam, UK), diluted 1:500, followed by the addition of Goat anti-mouse IgG (whole molecule) Peroxidase Conjugate (Sigma–Aldrich, USA) secondary antibody diluted 1:1,000, and detected using the GE Healthcare Amersham™ ECL™ Prime Western Blotting Detection Reagent (Thermo Fisher Scientific, USA). The Western blot was analyzed using an Azure Biosystems C300 (Azure Biosystems, Inc.) and cSeries Capture Software. Purified VLPs were analyzed using a Philips 208s Electron Microscope Morgagni (FEI, Czech Republic) at 18,000 × magnification and an accelerating voltage of 80 kV.

### BPV1 L1 identification and intact mass determination by MALDI-TOF

Excised protein bands of interest from Coomassie-stained 10% SDS-PAGE separating gels were subjected to a standard procedure of in-gel digestion (9) using SOLu trypsin (Merck, Germany). Peptides from the tryptic digests were purified using ZipTip C18 pipette tips (Merck) and separated in an nLC-MALDI system comprising an UltiMate 3000 RSLCnano liquid chromatograph (Thermo Fisher Scientific, Germany) and a Proteineer fc II fraction collector (Bruker Daltonik, Germany) (10). The eluted peptide fractions were collected on the target plate and directly co-crystallized with α-cyano-4-hydroxycinnamic acid as a matrix. MS and MS/MS data were then acquired on an ultrafleXtreme MALDI-TOF/TOF instrument (Bruker Daltonik) (10). Protein identification was achieved using flexAnalysis 3.4 and ProteinScape 3.1 software (Bruker Daltonik) plus Mascot Server 2.4 (Matrix Science, UK) by searching the MS/MS data against the Swiss-Prot database (Release 2022_04).

### Immunization of mice

Six-week-old female BALB/c mice were purchased from the Laboratory Animal Breeding and Experimental Facility, MUNI, Czech Republic. Experiments were conducted according to the principles enunciated in the Guide for the Care and Use of Laboratory Animals issued by the Czech Society for Laboratory Animal Science. The animal experiments were reviewed by the ethical committee and approved by the Ministry of Agriculture of the Czech Republic (permit no. 9487/2019-3). Mice were divided into four groups (*n* = 4/group) as follows:

1. Group immunized with 10 μg purified VLPs in sterile buffer [50 mmol l−1 Tris-HCl, 300 mol l−1 NaCl, pH 7.4], supplemented with 1% saponin (Merck, Germany) and 15% alhydrogel (Merck) per dose as adjuvants.
2. Group immunized with 25 μg purified VLPs in a sterile buffer with adjuvants.
3. Group immunized with 50 μg purified VLPs in a sterile buffer with adjuvants.
4. Control group injected only with a sterile buffer.

To obtain VLPs-specific hyperimmune serum, mice were immunized two times subcutaneously with a booster 3 weeks after the first immunization. Blood was harvested before immunization and 3 and 6 weeks after the first immunization.

### ELISA analyses

An ELISA plate was coated with 1 μg purified BPV L1 VLPs or native BPV1 virus isolated from organ suspension and incubated for 2 h at 37°C. After washing three times, the plate was blocked by 5% non-fat milk and incubated for 30 min at 37°C. The plate was washed three times, and 100 μl mice sera diluted 1:400 was pipetted onto the plate and incubated overnight at 4°C. After washing, Goat anti-mouse IgG (whole molecule) Peroxidase Conjugate (Sigma–Aldrich) in a dilution 1:8,000 was pipetted into each well of the plate and incubated for 2 h at room temperature. After washing six times, 100 μl TMB substrate (INgezim Circo IgG; Eurofins Technologies Ingensa, Spain) was added and incubated at room temperature. The reaction was stopped using 100 μl stop solution, and the optical density was analyzed at 450 nm using Gen5^TM^ Data Analysis Software (BioTek, USA).

### Statistical analysis

The non-parametric Kruskal–Wallis test with Dunn's multiple comparisons test was used to compare differences between the groups of non-immunized mice and mice immunized by purified BPV1 L1 protein at different doses. A *p* < 0.05 was considered statistically significant.

## Results

**Figure 1:**
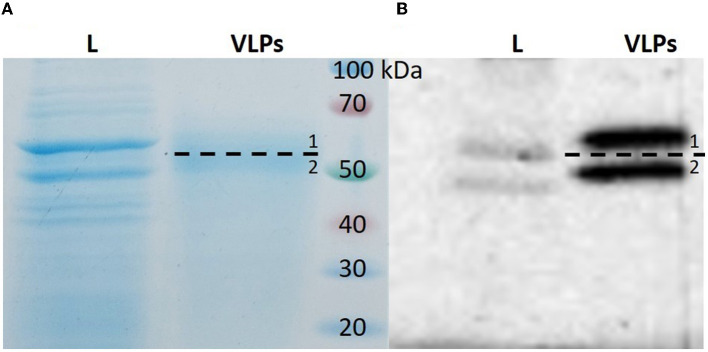
SDS-PAGE and Western blot analysis of purified BPV1 L1 VLPs. Purified protein was resolved on 10% SDS-PAGE followed by Coomassie brilliant blue staining **(A)** or analyzed by Western blot **(B)**. Two specific bands were identified (1—top band, 2—bottom band). L, cell lysate; VLPs, purified protein.

BPV1 L1 VLPs were produced in SF9 cells and purified by affinity chromatography using Lentil Lectin-Sepharose 4B. The purity of BPV L1 VLPs was analyzed by SDS-PAGE and Western blot with specific primary antibodies against BPV1 L1 protein (Figure 1). From 150 ml of cell culture (~0.75 g of cell pellet), we obtained 0.2 mg of purified BPV L1 VLPs.

**Figure 2:**
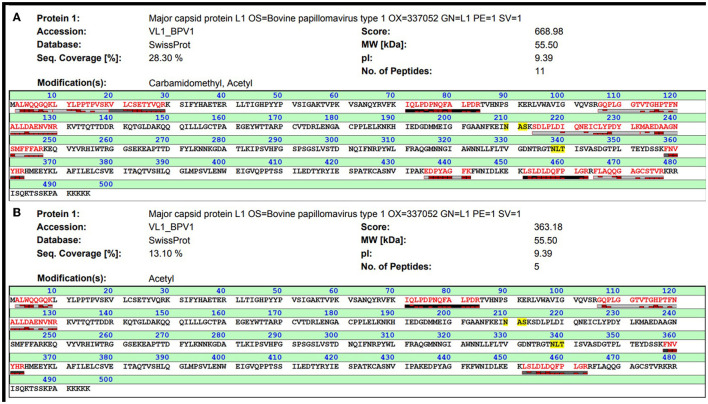
MALDI-TOF analysis of purified BPV1 L1 after in-gel tryptic digestion. Both protein bands separated by SDS-PAGE (see Figure 1) were processed for in-gel digestion, and the obtained peptides were analyzed by nLC-MALDI-TOF/TOF MS/MS. The identification results for the top and bottom bands are shown in **(A, B)**, respectively.

**Figure 3:**
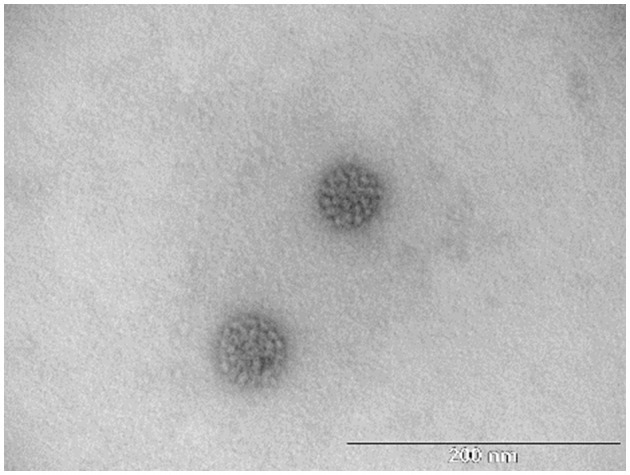
TEM micrographs of the isolated BPV1 L1 VLPs. Scale bar = 200 nm.

The two protein bands of purified BPV L1 visible in gels after SDS-PAGE and positively detected by Western blot (Figure 1) were processed for in-gel digestion and protein identification using nanoflow liquid chromatography coupled offline to matrix-assisted laser desorption/ionization time-of-flight/time-of-flight tandem mass spectrometry (nLC-MALDI-TOF/TOF MS/MS). The presence of BPV L1 was confirmed in both cases by assigning 5–11 peptides, providing probability-based score values of 360–670 (Swiss-Prot accession no. VL1_BPV1; Figure 2). The corresponding two protein forms differ slightly in their molecular mass and were found to be acetylated at the N-terminus, which may increase their stability against proteolysis (11, 12). The observed mass difference could arise from a partial truncation at the C-terminus, as the presence of a tryptic C-terminal peptide FLAQQGAGCSTVR was not general in the digests, or from a glycosylation heterogeneity (two potential *N*-glycosylation sites are present in the sequence). We confirmed the correct assembly of VLPs using electron microscopy (Figure 3).

**Figure 4:**
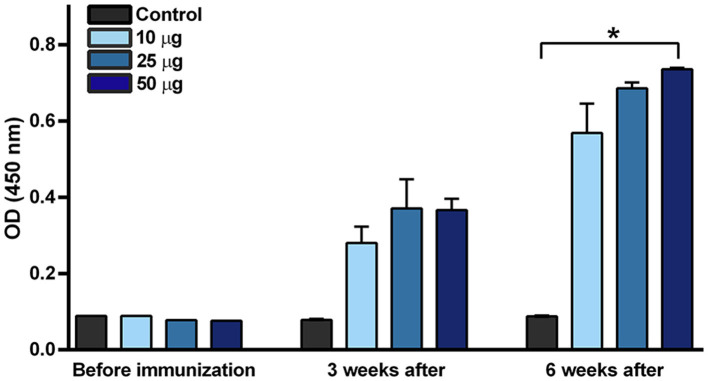
BPV1 L1-specific antibody immune response induced by purified VLPs. Three groups of mice were immunized two times (booster administered 3 weeks after the first dose) by purified VLPs supplemented with 1% saponin and 15% alhydrogel per dose as adjuvants, at concentrations of 10, 25, and 50 μg purified protein. Mice sera were collected before immunization and 3 and 6 weeks after the first immunization. Proportionally mixed sera of non-immunized mice were measured as controls before vaccination. *p < 0.05 vs. non-immunized control at 6 weeks. Mean ± SEM, *n* = 4, Kruskal–Wallis test with Dunn's multiple comparisons test.

Purified VLPs were sterilized by filtration and used for the vaccination of mice. Humoral immunity response was tested by ELISA pre-coated with BPV1 L1 VLPs (Figure 4). Increased levels of specific antibodies were detected in all groups of mice after the first immunization, with no significant differences between doses. The levels of specific antibodies markedly increased after the second immunization with a significant immune response in mice immunized with 50 μg purified VLPs. Similar results were obtained by ELISA pre-coated with BPV1 virus purified from an organ suspension of infected cows (data not shown).

## Discussion

No effective therapy is currently available for bovine papillomatosis or sarcoid tumors in horses. Traditional attenuated or killed vaccines from cultured BPVs have not been shown to be sufficiently effective. In recent years, BPV vaccines based on VLPs have been developed. These subunit vaccines contain one or more viral structural proteins that are capable of self-assembling into structures mimicking native viruses but without viral DNA (13, 14). They are structurally similar to native virions and can highly stimulate both humoral and cell-mediated immune responses (15). Cell-mediated immunity plays a particularly important role in the immune response against papillomavirus. The high protective effectiveness of VLP L1 vaccines comes from the ability of VLPs L1 to induce strong Th cell-dependent B-cell response associated with the production of high titers of neutralizing antibodies and B-cell memory (16). The prophylactic efficacy of VLP L1 may also be associated with the response of cytotoxic T lymphocytes to VLP L1 and the production of effector memory T cells (CD8+ T_RM_) (17–19). Long-term protection of VLP L1 immunized animals against BPV1 is also provided by the production of neutralizing antibodies aided by immune memory of plasma cells and memory of B and T cells (20). A mouse model is an essential tool for testing the mechanisms of immune response to vaccines. The accuracy in predicting how well the vaccine would work in target animals is often not quite optimal. This model has been successfully used for testing of L1 VLPs vaccines derived from BPV1, where BPV1 L1 VLPs vaccination completely protected mice from challenges with BPV1 pseudovirus (21).

Several VLPs and subunit vaccines based on L1 and L2 proteins against BPV1 were produced in bacteria, yeast, insect cells, or plants (22–25). The baculovirus expression system is commonly used for the production of VLPs in large amounts, but requires several purification steps for the complete elimination of live viruses, viral and baculoviral contaminating proteins, and DNA. Since the complexity of the purification process markedly increases the overall cost of the final product, we tried to compare several methods to propose a rational strategy for the isolation of VLPs, meeting all the quality and purity requirements. Purification of VLPs by ultracentrifugation in a gradient of cesium chloride requires long ultracentrifugation times and may affect the morphological integrity, functionality, and antigenicity of purified VLPs (26). This method was used, for example, for the purification of VLPs containing the L1 protein of canine oral papillomavirus, L1 protein of BPV1 virus, or a combination of L1 and L2 proteins of BPV4 virus all produced in insect cells (13, 27, 28).

Ion exchange chromatography represents another purification strategy. In our case, utilizing this method did not result in sufficient purity of the final product (data not shown). This is in agreement with Lima et al. (29) and Yang et al. (30) who used anion-exchange chromatography supplemented with an additional method for the purification of VLPs to an appropriate purity. Affinity chromatography using Lentil Lectin-Sepharose 4B for the purification of glycosylated proteins represents an effective strategy for the purification of VLPs. Roder et al. (31) applied this method for the purification of S and F glycoproteins of the respiratory syncytial virus with high purity and without loss of antigenicity. With this method, we obtained highly immunogenic VLPs capable of inducing an antibody response in mice at doses starting at 10 μg VLPs. The amounts of IgG-specific antibodies were significantly increased after the booster, with the best response in mice vaccinated with the highest dose of antigen. Compared with the methods used so far, the uniqueness and advantages of the presented purification method are its simplicity and high effectiveness in obtaining highly pure BPV1 L1 protein, which could lower the overall price of vaccine production. Since cell-mediated immunity plays an important role in this process, our future studies will evaluate the effectiveness of our vaccine in cattle by monitoring the lymphocyte proliferation and measuring the cytokines in calves infected intradermally with BPV 1 virus.

BPV1 L1 VLPs prepared using BEVS and purified by Lentil Lectin-Sepharose 4B chromatography meet all the quality requirements for purity, stability, and biological safety, and represent a promising tool for prophylactic immunization against bovine papillomatosis and equine sarcoid tumors.

## Data availability statement

The datasets presented in this study can be found in online repositories. The names of the repository/repositories and accession number(s) can be found in the article/supplementary material.

## Ethics statement

The animal study was reviewed and approved by the Czech Society for Laboratory Animal Science reviewed by the Ethical Committee and approved by the Ministry of Agriculture of the Czech Republic (Permit no. 9487/2019-3).

## Author contributions

AV: writing—original draft preparation, investigation, and formal analysis. VB: investigation, formal analysis, and statistical analysis. IP: conceptualization, methodology, and funding acquisition. MFo and MS: investigation and formal analysis. MFr, PK, and RH: investigation. JM: investigation and funding acquisition. All authors have read and approved the final manuscript.
